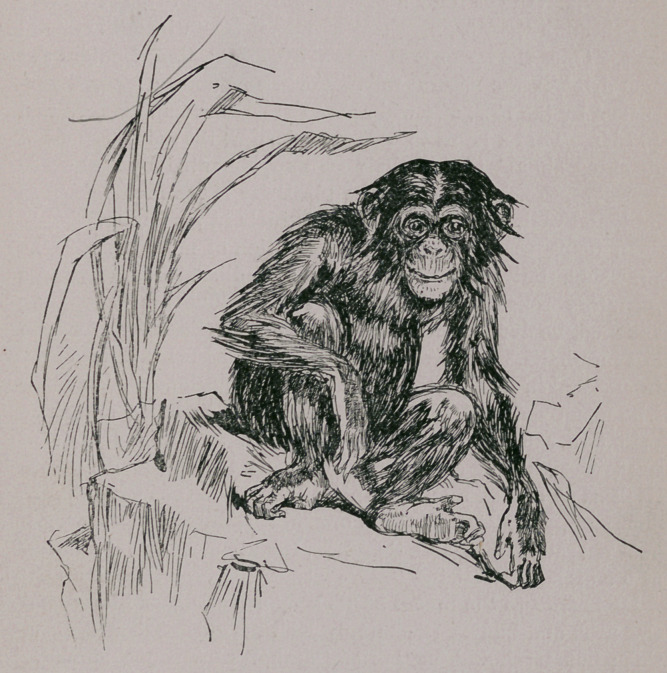# Notes, with Commentations, on the Psychology of the Chimpanzee, (Troglodytes Niger), Now in Captivity at the Central Park Menagerie, New York

**Published:** 1885-01

**Authors:** C. Pitfield Mitchell


### Art. V.—NOTES, WITH COMMENTATIONS, ON THE PSYCHOLOGY OF THE CHIMPANZEE (*TROGLODYTES NIGER*), NOW IN CAPTIVITY AT THE CENTRAL PARK MENAGERIE, NEW YORK.

BY DR. C. PITFIELD MITCHELL.

The following observations and experiments were instituted with a view more especially to adding, if possible, to the results of previous investigations of emotional expression in the anthropoid apes. This was, throughout, the ruling purpose ; yet whatever offered itself in the course of the inquiry, was recorded if it appeared to be of sufficient interest or of scientific value.

Elaborateness has been given a secondary place to accurateness of observation. Many of the experiments were several times repeated, and other opportunities were used for the correction of errors and the filling in of omissions. Owing partly to the transitoriness of the phenomena observed, there is, doubtless, still room for further purification as well as for further amplification.

I am much indebted to Dr. W. A. Conklin, the Superintendent of the Menagerie, for very valuable assistance and liberal courtesy.

The subject is a male chimpanzee, now (November, 1884) about two years old. He arrived in this country from Liberia, West Coast of Africa, in June of the present year.

Sept. 30, 1884.—On being introduced he offers his right forehand, and grasping one of my fingers, attempts to put it in his mouth. [He appears to enjoy squeezing the fingers of his friends between his teeth. But this ought not, I think, to be understood as simply an expression of attachment. He does, it is true, show affection by kissing and licking; yet it is his constant habit to interrogate the nature of anything by putting it to the tests of teeth, tongue, and lips. The very mobile and prehensile lips of the chimpanzee are probably in constant use as tactile organs, and he is plainly much swayed by considerations concerning the edibleness of things; therefore, whatever comes within the range of his prehension is instinctively carried to the mouth. The same proclivity has been frequently observed in infants.

The extension of the hand, on meeting an acquaintance, is made with a pleased look of recognition, but without perceptible contraction of facial muscles, and is unmistakably the outcome of gratified social feeling. On these occasions the body is sometimes couched submissively and the back is turned in solicitation of a friendly scratching. The hand is offered to any one towards whom he is disposed to be friendly.]

Being placed by Mr. Jacob Cook, his keeper, in a chair before a table upon which lie a bowl of milk, a spoon, and a napkin, he grasps the spoon with his right fore-hand and feeds himself, wiping his lips with the napkin held in the left fore-hand. [These super-simious table manners have been acquired, Dr. Conklin says, with singular aptness, under the tuition of Mr. Cook. In the feral state the chimpanzee drinks by sucking through the lips, precisely as man drinks without artificial aids. Once the spoon was taken from him, that I might see him drink in the natural way. For a moment he seemed perplexed by the loss, and whimpered to have the spoon returned to him. That a habit so well organized as the natural mode of drinking should thus facilely be supplemented by a new adjustment, exemplifies again the plasticity of instincts * in general, and perhaps of anthropoid instincts in particular.]

In using the spoon the co-ordination of movements lacks precision, but none of the milk is spilled. He expresses gustatory relish by smacking the lips. The appetite being satisfied, he denotes the fact by slapping the table with the left fore-hand. [This action is habitual, and is not improbably intentional in that he thus attracts the attention of his keeper who usually after feeding him takes him in his arms. The primitive cause of the action, perhaps, lies in a feeling of uneasiness, having completed something that no longer offers itself as an object upon which he can expend his super-abundant energy. Restlessness is one of the chimpanzee's prominent characteristics, and, therefore, Impatience may be inferred as a co-relative trait.]

He nestles to the breast of his keeper and clasps him round the neck and body. [Seemingly he thus demonstrates affection; but it should be borne in mind that young monkeys embrace their parents in this way for protection and for transportation. He has been noticed to make the movement with the eagerness of anticipated pleasure. It may be supposed that during the process of evolution, this close bodily union would, from associated conditions in the organism and

* Cf. "Mental Evolution in Animals," by George John Romanes, M.A., LL.D., F.R.S.

its environment, give birth to and become welded with feelings of large mass and momentum; and possibly, I think probably, here may be discerned one of the utilitarian origins of embracing as a mode of expressing strong attachment among ourselves.]

An opportunity was now given to observe the facial, and other outward signs, of a general feeling of Pleasure, compounded of voluminous simple-sensation—pleasure, the pleasure of play, and perhaps the pleasure attending requital of affection.

Taking the chimpanzee upon his knees the keeper tickled him about the armpits and playfully fondled him.

Moderate enjoyment was manifested by a slight parting of the lips and opening of the mouth, and by quick movements of the limbs and trunk in flexion. Stronger feeling by a broad grin, displaying both rows of teeth—the mouth widely open—and by moderately loud, low-pitched chuckles; closely simulating the vocal elements of human laughter. The excitations of the *risorii* and *zygomatici*, especially the latter, are well-marked; the angles of the mouth being drawn outwards and conspicuously upwards. [The observation above, respecting the exposure of teeth, slightly conflicts with an observation of Mr. Darwin. He remarks:* "The teeth in the upper jaw of the chimpanzee are not exposed when they utter their laughing noise, in which respect they differ from us." Repeated examination established the facts that in laughter of moderate degree, only the teeth of the lower jaw are uncovered; but in laughter of considerable intensity the teeth of both lower and upper jaws are well exposed.

It was not observed as a result of the experiment recorded, nor subsequently, that the eyes and the muscles about the orbits gave any recognizable indications of the inward feeling. These parts of the face are generally expressionless. During laughter the eyes are perhaps less immobile than usual, but the eye-lids are quite open, there is no perceptible lachrymation, and the face is not suffused with blood. The naso-labial fold of infantile and adult laughter is plainly marked. When the volume of pleasurable sensation is small, the lips are separated with an expression that may properly be called a smile.

* "The Expression of the Emotions in Man and Animals," page 133. Appleton & Co., New York, 1873.

In the chimpanzee then the outward and visible signs of laughter are comparatively simple. Still, it was demonstrated that the energy set free seeks escape not only along some of the same tracks of neuro-muscular tissue as in simple laughter in man, but that a relation, as in man, subsists between proportions of liberated energy and particular avenues of outlet. If the quantity of feeling is small, the lines of discharge terminate in labial muscles—the lines made most permeable by constant use; if the quantity is large, the labial muscles become more strongly contracted, and the energy overflows through respiratory, vocal, and voluntary neuro-muscular systems. The contractions of voluntary muscles, however, in the instance related, are not considerably of central origin, but are in larger measure reflexes from the cutaneous periphery.

That species of laughter which is caused by the perception of certain incongruities,* as a clown in female clothing, a parrot uttering oracular speeches, was never witnessed in the chimpanzee, though two or three attempts were made to evoke this kind of risibility. The facts of comparative psychology and the doctrine of evolution would invite expectation of these negative results. A true sense of the ridiculous,† the laugh of scorn—derision, the ironical laugh, the smile of pity, are specialized differentia of the expression under examination, and their evolution would be contemporaneous with the unfolding of a higher grade of cognitive faculty than is found in any of the lower animals.

The resemblance between the physiognomic language of laughter due to simple-sense pleasure, and the physiognomic language of anger, is, as succeeding notes will show, very striking; especially is it so when we consider the apparent antithesis of the subjective accompaniments. Sometimes the resemblance makes it doubtful whether the chimpanzee is pleased or displeased. In both cases the mouth may be open, its corners retracted, and the teeth exposed. And there are many other likenesses quite deserving of exhaustive consideration from the standpoints of physiology and evolution.]

---

*See Herbert Spencer's essay "The Physiology of Laughter."

† Not a sensitiveness to ridicule, which dogs and monkeys manifest.

We were afterwards to note the chimpanzee's behavior under a feeling of Displeasure with Disappointment as one of the root-elements.

A piece of banana, of which fruit he is very fond, was offered him by his keeper through the bars of the cage, and withdrawn as he approached to receive it. At first he sulked with pouted lips, and then uttered prolonged squeaks, drawing the upper lip tensely over the upper row of teeth. As the keeper essayed to go away with the banana, he became angry, crying with mouth widely open and the angles drawn outwards. Both rows of teeth were exposed. He next retired from the front of the cage, and standing upright with his back to one side of it violently shook the hands, after the manner of very young children under similar circumstances.

The banana being given to him, the face assumed a contented but placid expression.

In putting the banana through the bars of the cage, a small piece of the fruit was left upon the outside of an external wire netting, and to obtain this piece the right fore-finger and prehensile lips were employed with nicest co-ordination of action. He clears his nostrils by scooping in the cavities with the tip of the fore-finger.

October 1st, 1884.—To-day the chimpanzee is less vivacious and is disinclined to indulge us in our wishes to elicit his mental qualities. When an attempt is made by the keeper to incite him to jealousy, by nursing with assumed tenderness and affection a monkey (*Cercopithecus griseo-viridis*) which has just died, he looks on with stolid indifference. The dead monkey being placed within his reach he examines it with an intelligent but silent scrutiny. Evidently he recognizes the monkey as one of his kind, and in doing so he employs the sense of smell as well as the sense of sight; but he thinks it is sleeping, for he tries to arouse it by pressing his knuckles into its flesh and by pulling its hair. If he has a distinct perception that the monkey is dead, this perception is without strong emotional associations, inasmuch as he comports himself with perfect impassivity; for assuming, without the warrant of fact, his ability to control the signs of feeling, there was not in the occasion any motive for concealment of feeling.

A little dog is placed not quite within his reach. He observes it with a quiet air, touching it with his fingers. Seizing a change to grasp it, he squeezes one of its limbs between his finger and thumb, betraying the accompanying feeling by firm compression of the lips—contraction of the orbicularis oris.

[We ourselves compress the lips in this way under various kindred emotions. Generally these emotions are counterparts of organismal actions determined by resistances in the environment, or cognized as resistances. There may be instanced the features in determination, implacability, hostility, incipient anger, and the firm closure of the lips in some people when thinking laboriously. In these cases the physiological condition is an energizing of the higher parts of the brain to overcome opposing forces, and this naturally expresses itself, in general, by muscular tension. In converse cases, where the cognition is of neutral or attractive forces, muscles are relaxed; as we see in ease, contentment, the smile of pleasure, the look of love, the attitude of devotion.]

October 3d, 1884.—A kitten was put in the cage with the chimpanzee. He took it up playfully and held it at first with much gentleness and even tenderness, but afterwards took it by the tail and swung it round several times. He then carried it across the cage, swinging it by one of its limbs, at the same time grinning, the mouth being open and the teeth exposed, with a cruel and wanton expression of countenance. The kitten was then taken from him. It was not positively observed whether the teeth of both upper and lower jaws were uncovered.

[We are reminded by the foregoing incident of the behavior towards dumb creatures of children before the growth of the sympathies. Babies and very young children are wont to seize kittens and puppies by an ear, a limb, or the tail; and it is not until there is some mental representation of the suffering thus caused and the danger incurred of being bitten or scratched, that animals are handled with gentleness and care.

That tender emotions are experienced may be inferred from the fact that he pressed the kitten to his breast and kissed it, holding it very gently in both hands. In kissing, the lips are pouted and the tongue protruded, and both are pressed upon the object of affection. The act is not accompanied by any sound, thus differing from ordinary human osculation.

The smile of cruelty we may interpret, from the evolution point of view, as the facial index of feelings arising from the exercise of consciously superior power.*]

When matches were lighted before him, at the moment of ignition he started back, spasmodically closing the eyelids. Curiosity as to the nature of the light led him to put his finger in the flame, and then place his finger to his nostrils. He burnt his finger slightly and placed it for a moment between his lips in contact with the teeth and tongue.

We now watched the effect of introducing him to his reflection in a large mirror.

Peering round the edge of the glass, he perceived his image, and remained for a second fixed with surprise. He then looked to the back of the mirror, and began to bite the frame and pull an attached cord as if in anger. Advancing to the front and examining the reflection of his person with evident satisfaction, he commenced, with absurdly sincere intentions, to make effusive demonstrations of love. He repeatedly pressed his lips and tongue to the glass, and erecting himself to his full height, strutted, and grinned, and made obeisance in most ridiculous and amusing fashion. He was once seen to make signs to his image by spasmodic movements of the lips, without uttering any audible sound. He again looked behind the mirror, and again fell to biting the frame. He became still more angry, and hit the glass, first with the left fore-hand and then with the left hind-hand, and continued to do so with such violence that we were finally compelled to break the spell.

While eating some fruit he saw himself in the glass, and ran away precipitately that he might keep possession of his morsel. [This rather trite experiment serves to show us the simplicity of his processes of Recognition. They are not marked perspicuously by the conscious comparison of relations, but partake somewhat of the nature of instincts in being relatively implastic. It is true that he looked behind the mirror, but he did so, I think, from a desire for contact with the supposed monkey, and not to verify visual perceptions by actual perceptions; for the complete contradictory evidence from the latter source did not in any noticeable degree modify the

* Cf. Mind; "A Classification of Feelings," by C. Mercier, M.B., July, 1884.

precept derived from the former: he continued to be satisfied of the validity of his first cognition.

His violence towards the mirror may be construed as the outcome of unsatisfied desire, or as active resentment towards an offending object; and in the latter case recalls the instinctive disposition in some children to vent spleen upon inanimate things through which they have sustained hurt.]

Additional and check observations were made of the chimpanzee's conduct under antagonistic feeling. A portion of an orange, the paring of which he had eagerly watched, was placed in a cigar box by his keeper, instead of being handed to him according to his anticipation. He thereupon gave forth a faint, whining, murmuring cry, his head was depressed, i.e., flexed upon the thorax, his eyes were sullen in expression, and his lips forwardly pouted, and he retreated from us. On a like occasion the interesting fact was noted that he sought solitude under a table, turning his back towards us, with head to the ground.

The experiment was repeated, with this modification, that in place of the keeper I was the person withholding the piece of fruit. Now he screamed loudly; the mouth was opened, its angles retracted, and both rows of teeth uncovered. The hands were shaken vigorously; the entire ebullition of feeling being highly suggestive of, and, indeed, only to be adequately described by reference to the crying of an infant.

The piece of fruit was withheld from him a third time, again by me, and to the effect that he gave way to a fit of anger, passing into fury. He grinned with savage looks, danced about, stamping his feet, and with his hands cast upon me the saw-dust from the floor of the cage.

[Here we have, evolutionally considered, three distinct modes of opposition to counter-forces in the environment. As suggested by the very important classification of feelings recently discovered by Dr. Charles Mercier ("Mind," loc. cit.) the first-described form is a passive antagonism—sulkiness; the second, active-passive—crying with vexation; and the third, active—anger and rage. But though these three varieties of feeling had the distinctive characters described, stages of transition from one to the other were noticed when the provoking influences underwent changes of amplitude.

Three or four observations lent support to, but did not quite confirm, the opinion that the chimpanzee's sulkiness is a passive protest against an unfriendly act of one to whom he is bound by ties of affection, or one from whom he is wont to receive favors, and therefore cannot actively resist. He was more inclined to be sulky or sullen when the fruit was refused him by his keeper than when refused him by others. He did, however, exhibit vexation or annoyance when the keeper showed much persistency, and on later dates cried when opposed by him; but crying is not retaliatory. It would seem, therefore, that wounded-feeling is one of the constituents of sulkiness, and gives to it the quality of passiveness. These suppositions are strengthened when we consider that one of the essential conditions of sulkiness in other cases, is some degree of social union and social concord. We may be angry, contemptuous, or timorous towards an enemy, or a rival, but not sulky.

The movement of the body *away* from the offending person, and the turning of the back to him, with depression of the head, are clearly anti-social in meaning, and were present, more or less markedly, with every exhibition of sulkiness. That the chimpanzee should seek solitude, as mentioned above, is very interesting when we bear in mind the etymology of our words sullen, and, probably, sulky: O. Eng., *solein, solain*, lonely, sullen; Lat., *solus*, alone. Why are the lips pouted in sulkiness? If the foregoing inference concerning the composition of this state of mind be true, namely, that one of its components is disappointed affection, it seems not inconsistent with the principle that nervous energy tends to discharge itself through familiar channels, that the special and complex feeling originated should express itself as tender emotion is expressed, by pouted lips. Mr. Darwin thinks, and arrays numerous and cogent facts as the ground of his opinion, that the pouted or tubular conformation of lips is assumed for the production of the whining noise generally accompanying displays of sulkiness. And unquestionably this is partly the explanation, but not, I think, wholly. As negatively supporting the view here put forth, I have heard the chimpanzee make the same whining sound with the crying expression of face; and as positively supporting it, it may be stated that children sometimes pout in shyness, a state of mind *sui generis* in its dependence upon a social relation of special quality. In all probability two or more co-operative causes have been at work in the evolution of pouting as an emotional expression.

Special attention was given to ascertaining whether tears are excreted in crying. I cannot assert that there is *no* increase in the lachyrmal secretion, but an easily observable increase does certainly not take place. The eyes are not closed as in children, and therefore one of the causes of shedding tears in crying is absent. As many are aware, infants of very tender years, less than two to four months old, cry without weeping. Mr. Darwin says of his own determination of the point, "I first noticed this fact from having accidentally brushed with the cuff of my coat the open eye of one of my infants when seventy-seven days old, causing the eye to water freely; and though the child screamed violently the other eye remained dry, or was only slightly suffused with tears." To the evolutionist this tearless crying of infants and chimpanzees is a coincidence of facts of considerable interest.

When young children cry the mouth takes a somewhat square outline from the angular portions of the lower lip being drawn downwards by the *depressores anguli oris*, and from the same portions of the upper lip being drawn upwards by the *levatores anguli oris* and *zygomatici*. As Mr. Darwin quotes of a child that was fed while crying, "it made its mouth like a square and let the porridge run out at all four corners."

This contraction of the *depressores anguli oris*, the chimpanzee when crying does not display in common with children, so that the mouth has not the squarish form; the angles of the mouth are drawn simply outwards and upwards. Twitching of the depressor muscles and sobbing usually terminate a fit of crying in older children. Infants do not sob. In the case of the chimpanzee the fit ends according to the incident conditions, abruptly, or with pettish whining. The quick, short, spasmodic inspirations succeeding the prolonged expirations of infantile crying, though looked for, were not seen.

The superior varieties of mankind seldom cry or weep; and in all likelihood the power to control the signs of suffering has been acquired quite recently in the history of the order Bimana. Some savages cry in the manner and from the causes prevailing among children. In certain disorders and diseases of the nervous system, as temporary depression of the vital powers from whatever cause arising, debility, paralysis, and cerebral softening, tears are drawn with pitiful readiness. And "weeping is common in the insane, even after complete fatuity has been reached and the power of speech lost." These facts are supremely interesting from the standpoint of the complementary doctrine to Evolution—the doctrine of Dissolution. The latter embodies the truth that, if the conditions are favorable, the unbuilding of the organism by the destructive agencies of its conditions, is in the opposite order of its building during the vast cycles of evolution; and also implicates, as an accessory, the truth that the later evolved functional and structural attributes of the mind and body, are less stable or enduring than those antecedently evolved. By the illumination of this conception we are enabled to understand why the mental capacities by means of which the expressions of pathetic feeling are suppressed—possessions won by the race but as yesterday—are insecure and fleeting as compared with the capacities for showing suffering, which were organized probably during inconceivably remote eras. Further, speech, which is but the "language of the emotions" elaborated, appeared during evolution later than this language, as the chimpanzee eloquently testifies, and disappears, during dissolution, sooner, as the insane so pathetically testify.

In connection with the principle of dissolution it may be mentioned that during convalescence from an illness from which the chimpanzee suffered at a later date, there was, as we observe in ourselves under like conditions, loss of control over aggressive feeling.

When very angry the chimpanzee will sometimes throw himself upon the abdomen, throw his arms wildly about, strike the floor, and bite those who touch him, just as children often do. The ears are expressionless in anger as in other emotions; but, having attended to the point with some care, I can affirm, contrary to the observations of others, that the pinnæ do move slightly as a whole, as if from contraction of their intrinsic muscles and not from tremor of their flexible cartilages. The face is not distinctly reddened.

If we contrast crying and anger in the case of our subject,

ARMY MEDICAL LIBRARY
WASHINGTON, D. C. U. S. A.

with crying and anger in the case of children, we find, as we found in laughter, that the anthropoid emotions are characterized by greater homogeneity, simplicity, and indefiniteness, in harmony with evolutionary truth.]

October 7th, 1884.—A colored india-rubber ball, making a musical note when the contained air was expelled, was shown to him, and the sound produced. He started away, looking a little alarmed, and then advanced and visually examined the object with an air of curiosity. When the ball was given to him to play with he was evidently doubtful of its harmlessness, and touched it rather gingerly, rolling it about the cage. At length he took the ball in his hands, not seeming afraid, and tried by gentle pressure, in imperfect imitation of what he had seen me do, to evoke its note. Failing in this, he commenced to hit it forcibly with the knuckles, and grinned with pleasure when the sound was produced. He then hit it violently, drawing the upper lip over the upper row of teeth, looking as if delighted in the exercise of his powers.

He was allowed to see a piece of fruit put in a tin box or canister, and the latter closed by a firm adjustment of the lid. He very quickly applied the teeth, not the fingers, to remove the lid, and having succeeded in doing so, extracted the fruit. But seeing a similar cover on the opposite end of the canister the previous association of contiguity between an adjusted cover and enclosed fruit, forced him unreasoningly to remove this cover also.

A pretence was made of putting the fruit in the box, the lid being applied as before. When he found that the box was empty his face assumed a very comical blank look of checked expectancy. The hand was inserted three or four times into the box and the latter held in position for ocular inspection. He then took off the remaining cover and, still unconvinced that there was no fruit, held the canister to his eyes, spying through it from end to end, and repeated this action more than once. [It is worth while marking the fixedness of the animal's prepossession. The pleasing belief that there was fruit in the box, for a time rendered cognitions from the most trustworthy senses—impressions entirely inoperative in the formation of a new conviction.]

When trying to extract the piece of fruit by inserting the hand, which the box was just large enough to admit, the lips were slightly compressed, as we compress our lips in acts necessitating nicety of muscular adjustment—for example, learning to write, untying a knot, etc. Attention has been drawn to a general quality, or condition of the correspondence between the organism and its environment required to call out this expression

October 13th, 1884.—A painted terrapin (*clemmys picta*) was placed in the cage with the chimpanzee. He stood on all fours at a distance of several feet from it, and eyed it with strained and fearful attention. The mouth was a little open, the teeth were not exposed. The upper lip twitched spasmodically, probably from contractions of the *levator labii superioris* and *zygomaticus minor*. As the reptile crawled away from him, he followed it, throwing saw-dust with the fore-hand, and stamping with the hind limbs as if to frighten his enemy. When the terrapin turned towards him he retired looking terrified. The face wore a distressed, not very definable, expression of extreme fear, and the hair over the entire body stood on end, falling again as the space increased between himself and the object of his terror, or as the supposed maleficent agent assumed an apparently less aggressive attitude. When Mr. Cook held the monkey with one hand, and the tortoise with the other, the former struggled actively to escape, and succeeding, sought security near the roof of the cage.

When the tortoise was taken away altogether, the chimpanzee sat upon a table breathing hurriedly; the limbs and features were visibly relaxed, the assemblage of signs being very expressive of Relief—the removal from consciousness of something perceived as imminently harmful. Dr. Conklin thought the face was paler than during the period of excitement, and I am inclined to corroborate the observation. Under changed circumstances, the terrapin and other harmless (though apparently to the chimpanzee harmful), animals were placed within his immediate environment. As noteworthy additional results, may be mentioned the studied caution with which he ventured upon a more intimate acquaintance with the fear-inspiring creatures. On one occasion, before touching the terrapin with one hand, he took the sagacious *pre*caution to hold open with the other hand the swinging door of his cage, that if necessary he might obtain safety within.

Nothing could more decisively demonstrate than these experiments the existence of natural inalienable affinities among Feelings, their insensible gradation one into the other, and the dependence of every grade upon quantitative and qualitative relations between the organism and the external world. When the terrapin or other animal was cognized as overwhelmingly maleficent, the outward signs were those of Terror; when cognized as simply dangerous, Fear and Timidity were the expressed emotions; when as possibly harmless, Confidence, Courage and Boldness were shown; when as certainly harmless and of insignificant power, there was manifested Cruelty; and when as attractive or beneficent—as in the case of a tame white rat—there was witnessed an exhibition of Tender Feeling.

October 15, 1884.—A child's harmonica was played before the bars of the cage and attracted his close attention. The instrument was given to him, and he at once placed it between his teeth in proper position for producing the sound, but afterwards in the wrong position. He now took the harmonica in his hands, examined it and shook it as if wishing to find out its nature. He knocked it upon the floor and hit it with his knuckles. When Mr. Cook played an air the chimpanzee was strongly influenced. He smiled broadly, the angles of the mouth being well raised, and patted his keeper upon the head with both hands, as if in the fullness of pleasure. He also applied his lips to the instrument. The rather surprising intensity of feeling shown may possibly be related to the feverishness from which the chimpanzee is suffering to-day, slight felicitousness being a common concomitant of the febrile state.

October 21, 1884.—A living monkey (*Cercopithecus cephus*) was held by Mr. Cook before the cage. The chimpanzee's upper lip became firmly compressed and pointed at the septum, with deep corrugations running from the latter feature outwards and upwards to the zygoma. He extended his hand and pulled the hair of the monkey's neck. The monkey attempted to bite him and squealed loudly. This appeared to make the chimpanzee angry. He hit the monkey, his face wearing a savage expression. He then made friendly overtures, patting the head of the monkey, pouting the lips as if wishing to kiss, and squeezing one of the monkey's feet between his teeth. The monkey resisted, with snarls and screams, and the chimpanzee then tried to beat it with the left hand, as if administering a merited punishment. This not availing as a corrective measure, he exposed his teeth, raising the upper lip and also the nose, after the manner of carnivora when they are enraged. This, I think, was done with the express intention of cowing the monkey, for he began to throw his arms about very wildly, with all the appearance of assumed passion; and, taking up a blanket lying at his feet, shook this in the air, evidently with the cunning purpose of frightening his antagonist into submission.

*(To be continued.)*

## Figures and Tables

**Figure f1:**